# Cyclic Behavior of U-Shaped Flexural Plates for Their Implementation in Multidirectional Energy Dissipation Devices

**DOI:** 10.3390/ma18081851

**Published:** 2025-04-17

**Authors:** Jorge González, Fernando Barriuso, Ramiro Bazáez, Luis Pérez, Gabriel Lara-Rodríguez, Rodrigo Astroza, Pablo Heresi

**Affiliations:** 1 Departamento de Obras Civiles, Universidad Técnica Federico Santa María, Valparaíso 2390123, Chile; 2 Departamento de Ingeniería Mecánica, Universidad Técnica Federico Santa María, Valparaíso 2390123, Chile; luis.perez@usm.cl; 3 Instituto de Investigaciones en Materiales, Universidad Nacional Autónoma de México, Ciudad de Mexico 04510, Mexico; laragab@unam.mx; 4 Facultad de Ingeniería y Ciencias Aplicadas, Universidad de los Andes, Santiago 7620086, Chile; rastroza@miuandes.cl; 5 Department of Civil Engineering, University of Chile, Santiago 8370449, Chile; pheresi@uchile.cl

**Keywords:** U-shaped flexural plate, fatigue, energy dissipation, multidirectional

## Abstract

U-shaped flexural plates (UFPs) are promising components for seismic energy dissipation due to their ability to undergo stable plastic deformation under cyclic loading. This study investigates their cyclic behavior through a combination of experimental testing and finite element simulations, focusing on their application in multidirectional damping systems. Key response parameters such as hysteretic behavior, energy dissipation, stiffness degradation, fatigue life, and the effect of loading direction were analyzed. The results demonstrate that UFPs provide reliable hysteretic behavior and maintain mechanical integrity over repeated cycles. The cyclic response was found to be strongly influenced by plate thickness, aspect ratio, and material yield strength. Based on these findings, this work proposes predictive equations for estimating strength, stiffness, fatigue life, and hysteretic damping of UFPs. Additionally, a simplified design procedure is presented for evaluating the strength and stiffness of multidirectional damping systems incorporating UFPs, with potential applications in bridges, buildings, and other structures exposed to complex seismic loading conditions.

## 1. Introduction

**Figure 1:**
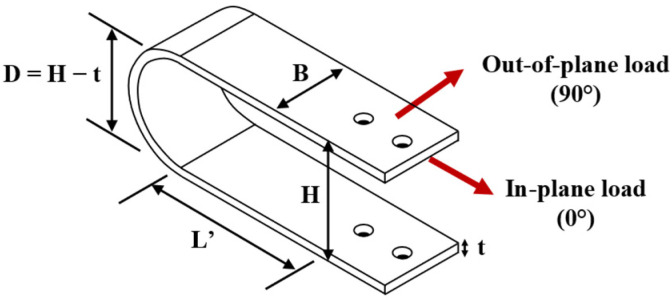
U-shaped geometry and bidirectional loading.

Seismic design increasingly relies on energy dissipation devices to protect structures by absorbing earthquake energy. These devices act as sacrificial fuses, yielding or deforming in a controlled manner so that the primary structural elements remain mostly elastic [1,2,3,4,5]. In bridges, which are critical components of transportation networks, such devices are especially important since preserving bridge integrity during earthquakes is vital for safety and post-event connectivity. Accordingly, modern seismic design codes and retrofitting strategies for bridges often incorporate passive damping elements (e.g., metallic dampers, base isolators, shock absorbers) to limit seismic forces and displacements. These energy dissipation systems have been used successfully in bridge bearings and connections to reduce vibrations, and they are becoming an integral part of resilient bridge design [6]. In that context, one promising type of metallic damper is the U-shaped flexural plate (UFP), which consists of a steel plate bent into a “U” shape that dissipates energy through flexural or torsional yielding of its arms as shown in Figure 1. The geometric parameters of UFPs are the overall height (H), width (B), plate thickness (t), diameter (D) of the semicircle, and length (L’) of the straight part without bolt holes.

Originally proposed by Kelly et al. in 1972 [1], UFP dampers have seen renewed attention in modern seismic design. For example, they have been used in coupled rocking shear wall systems and other applications [7,8], valued for their simplicity, low cost, and stable hysteretic behavior. Under cyclic loading, UFPs exhibit repeatable load–deformation loops with significant energy dissipation capacity. Because the curved U shape forces yielding to occur over a distributed region (essentially “rolling” plastic hinges), UFPs can sustain large cyclic deformations with stable strength. Several experimental investigations have validated the cyclic performance of UFPs [9,10,11,12,13]. Early tests in the 1990s examined U-shaped steel dampers in base isolation systems, characterizing their strength, stiffness, and energy dissipation under cyclic deformation [14]. More recent experiments include component tests on individual UFPs and assemblies: for example, Deng et al. [15] developed a “crawler” steel U-shaped damper for bridge applications and reported stable hysteretic behavior with significant energy dissipation capacity. Baird et al. [16] performed cyclic tests on UFPs of varying geometries to measure their initial stiffness, yield force, and post-yield response, confirming high ductility and providing data to calibrate design models. Current research continues to evolve with innovations in geometrical optimization and replaceability [17], hybrid configurations using shape memory alloys [18,19], and integration into novel applications such as post-tensioned mass timber systems [20] and energy-dissipating braces [21]. UFPs have also been successfully implemented in real structures. Notably, they have been used as replaceable dissipators in buildings (e.g., coupling beams in the Southern Cross Hospital in Christchurch [22] and in steel braces of buildings in Chile [23]) and have been considered and implemented in bridges as damping fuses or as supplementary energy dissipation for seismic isolation [24]. UFP dampers in bridges can be placed at expansion joints or between the superstructure and substructure to absorb seismic movements in lieu of conventional restrainers. Aldea et al. [6] recently conducted a comprehensive study on the implementation of bidirectional UFP-based hysteretic dampers in skewed multispan highway bridges. In their work, UFP dampers oriented to dissipate energy in both the transverse and longitudinal directions were incorporated into detailed 3D bridge models. Aldea et al. [6] demonstrated that these bidirectional UFP devices can markedly improve seismic performance in skewed bridges, thereby reducing the risk of span unseating and damage across a range of earthquake intensities. Their findings underscore the potential benefits of extending UFP applications from predominantly uniaxial uses (such as transverse-only dampers) to true multidirectional energy dissipation systems. Despite the vast research regarding UFPs, most prior studies have examined UFP dampers under uniaxial or in-plane bending conditions, whereas actual earthquake demands are often multidirectional [12,25]. There is a lack of research on UFPs used in multidirectional energy dissipation systems, such as devices that must concurrently restrain movement in both orthogonal horizontal directions. Furthermore, the low-cycle fatigue behavior of UFPs—i.e., their performance under repeated large inelastic cycles—has not been fully explored [26]. UFP dampers are expected to undergo numerous yield excursions during a major earthquake or across multiple seismic events, and their cyclic performance degrades with accumulating damage. Recent innovations, such as U-shaped dampers made from shape memory alloys, indicate that material enhancements can significantly improve fatigue resistance and show significant potential in addressing residual drift limitations in conventional metallic systems [18,27,28]. Thus, UFP fatigue life, stiffness degradation, and energy dissipation capacity under cyclic loading must be quantified when design-level and maximum considered (extreme) earthquakes are considered in a performance-based design framework.

Interestingly, while the UFPs are fabricated from solid isotropic steel or shape memory alloys, their configuration based on multiple elements connected through top and bottom plates bears a resemblance to sandwich-panel structures. These panels, widely used in aerospace and mechanical engineering, consist of two face sheets bonded to a lightweight core and are valued for their high strength-to-weight ratios [29]. Semi-cylindrical and honeycomb sandwich cores, inspired by bio-mimetic structures, have been developed for improved energy absorption and resistance [30]. The unintentional similarity between UFP assemblies and sandwich structures presents an interdisciplinary opportunity to explore lightweight configurations for seismic applications.

Although analytical formulations exist to estimate UFP yield strength and stiffness, there is still a lack of expressions to estimate hysteretic damping for different displacement ductility demands. Also, ensuring proper design requires understanding the device’s stiffness degradation and hardening, so that the primary structure can be capacity-protected accordingly. At present, there is no widely accepted simplified design method to account for these factors in UFP dampers, particularly when they are used in multidirectional configurations. Considering the above gaps, the present study aims to advance the understanding and application of UFPs in multidirectional seismic energy dissipation systems. The main objectives of this research are: (1) to investigate the cyclic behavior of UFPs subjected to different loading protocols and directions, through a combined experimental and numerical approach; (2) to characterize mechanical properties and low-cycle fatigue life of UFPs under cyclic loading; and (3) to develop practical expressions that enable engineers to estimate UFP damper strength, stiffness, and energy dissipation capacity for use in multidirectional systems.

## 2. Experimental Program

**Figure 2:**
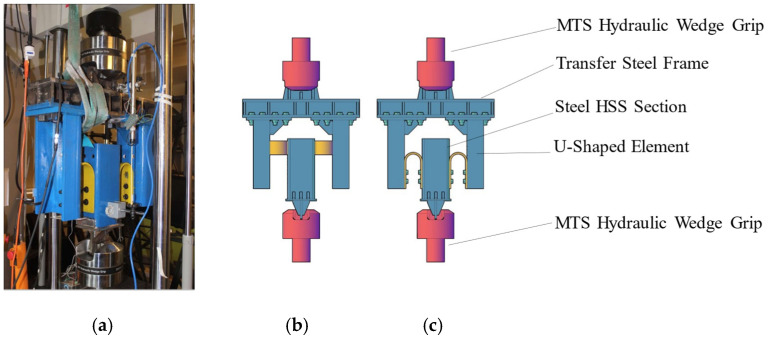
Test Setup (**a**) implemented in the laboratory, (**b**) schematic for loading at 90° and (**c**) 0°.

Forty-six (46) tests were carried out using an MTS 810 unidirectional testing machine powered by an MTS 505.30 hydraulic pump. A specially designed steel frame structure was installed on the testing machine to accommodate the UFPs. Figure 2 shows the experimental setup as implemented in the laboratory, along with a schematic diagram that clearly identifies each component of the testing system. Since UFP elements generate out-of-plane forces when deforming (as they tend to open laterally), a symmetrical system was developed to counteract and neutralize these forces. Consequently, two UFPs were utilized for tests conducted at 0° (in-plane), whereas four elements were employed for the 90° tests (out-of-plane).

The experimental matrix is presented in Table 1, using the following naming convention:

AXXX − {t or B or H} − {0G or 90G}

In this notation, AXXX represents the type of steel. The letters t, B, and H are used to indicate the thickness, width, and height of the specimen, respectively. Additionally, the designations “0G” and “90G” are used to specify the loading direction of the tests (see Figure 1). The test specimens were produced in a professional steel fabrication shop and manufactured using a cold forming process, in which steel sheets were bent at ambient temperature to achieve the desired curvature. The steel grades used in this study were ASTM A36 [31] and ASTM A572 Grade 50 [32].

### 2.1. Test Procedure and Instrumentation

**Figure 3:**
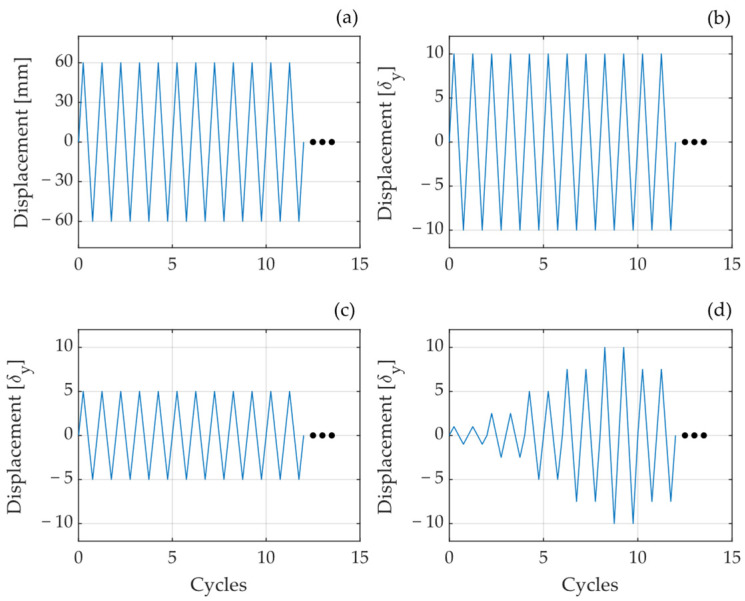
Loading Protocol. (**a**) Fatigue 1, (**b**) Fatigue 2, (**c**) Fatigue 3, (**d**) Increasing Amplitude.

Four loading protocols were selected to investigate the fatigue life of these elements, as shown in Figure 3. The Fatigue 1 protocol consists of cycles with a constant amplitude of 60 mm. Protocols Fatigue 2 and Fatigue 3 involve constant amplitude cycles equal to 10 and 5 times the yield displacement of the UFPs, respectively. The yield displacement ($\delta_{y}$) is calculated using Equation (1) proposed by Baird et al. [16], where $F_{y}$ is the yield force calculated using Equation (6). Lastly, the fourth protocol, referred to as Increasing Amplitude, is based on the loading protocol for the qualification of buckling restrained braces (BRBs) defined by AISC341 [33]. In this protocol, the design displacement was set as 5 times the yield displacement. Each of these protocols includes an indefinite number of cycles repeated until specimen failure occurs.

(1) $$\delta_{y}=\frac{27\pi F_{y}D^{3}}{16EBt^{3}}$$

**Figure 4:**
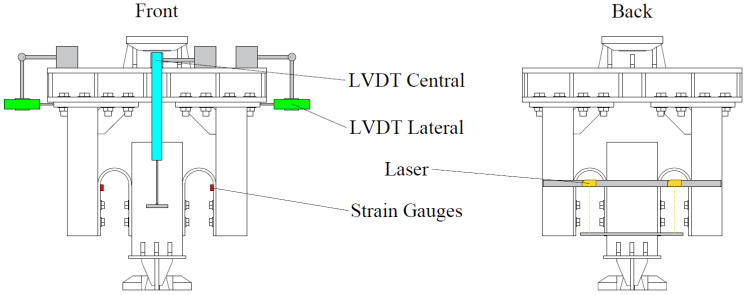
Instrumentation.

The instrumentation employed included the built-in sensors of the MTS testing machine, consisting of a linear variable differential transformer (LVDT) displacement sensor with a ±80 mm range and a load cell embedded in the upper grip, capable of measuring forces up to ±10 kN. Additional instrumentation, depicted in Figure 4, was also incorporated. A central LVDT with a ±100 mm displacement range was installed to measure the relative displacement of the UFPs more directly and accurately compared to the integrated machine sensor. Side-mounted LVDTs were included to monitor potential outward deformation or opening of the loading frame caused by out-of-plane forces generated by the UFPs. Furthermore, laser displacement sensors were positioned to provide redundancy in the measurement of relative displacement of the UFPs and to detect any possible rotation of the loading frame due to imbalances in specimen forces. Lastly, strain gauges were placed between the round and straight sections of the specimens to precisely determine the onset of yielding in these devices.

### 2.2. Test Results and Discussion

#### 2.2.1. Deformation Mode, Damage, and Failure

**Figure 5:**
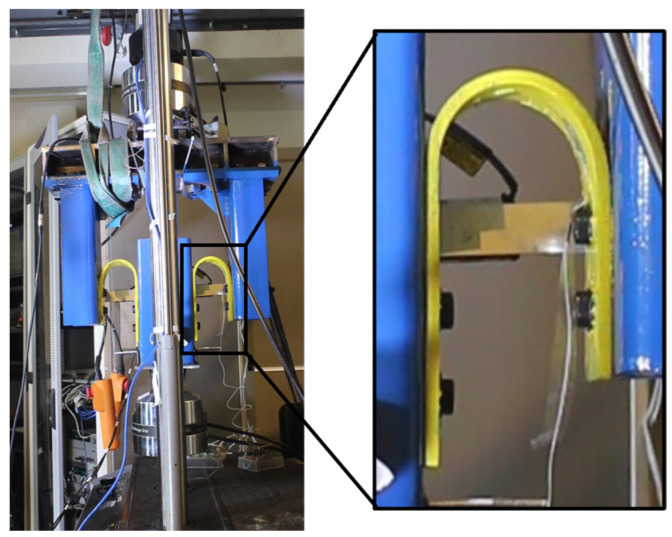
Deformation mode in specimen A572-0G-Fatigue1.

**Figure 6:**
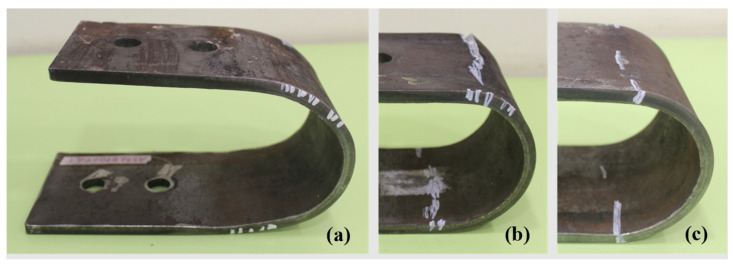
Crack patterns in specimens A572-B100. (**a**) Fatigue 1, (**b**) Fatigue 2, (**c**) Fatigue 3.

The deformation mode identified in the 0° tests is depicted in Figure 5, which clearly illustrates how UFP specimens deform during loading by opening outwardly against their constraints. This deformation mechanism results in plastic hinge formation at the junction between the curve and straight sections of the UFPs. As cyclic loading progresses, cracks initiate and propagate within these plastic hinge regions, as shown in Figure 6. Additionally, this figure clearly illustrates the correlation between the extent of cracking and the amplitude of the displacement cycles applied in each loading protocol. Specimen failure was ultimately defined either as the occurrence of fracture or as the moment when the load-carrying capacity dropped to 80% of its maximum recorded value.

**Figure 7:**
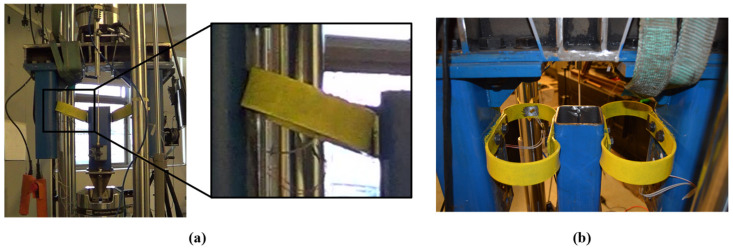
Deformation mode in specimen A36-B40-90G-Fatigue1. (**a**) During a loading cycle and (**b**) At the end of the test.

**Figure 8:**
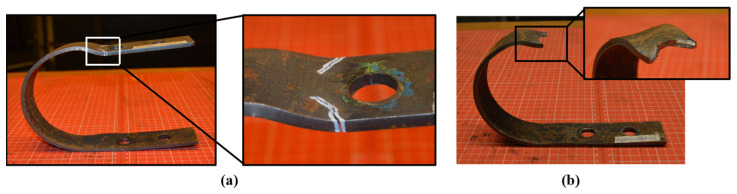
(**a**) Crack patterns and (**b**) Fracture of A36-B40-90G-Fatigue2.

In the case of loading applied at 90°, the deformation mode observed is illustrated in Figure 7a, showing how the deformation involves torsional plastic hinges forming in the region connecting the straight and curved sections of the UFP. Additionally, Figure 7b reveals out-of-plane deformation caused by the absence of lateral infinite planes that would ideally restrain the UFP elements. As cycling continued, cracks appeared in regions where torsional plastic hinges formed. These cracks were clearly associated with repeated cyclic loading. Figure 7b further demonstrates an out-of-plane deformation due to insufficient lateral support, emphasizing the necessity of lateral restraints for preventing undesired behavior. Eventually, after a significant number of cycles, cracks developed into semicircular fractures, as depicted in Figure 8. This distinctive semicircular fracture pattern signifies the ultimate failure mode of specimens subjected to out-of-plane loading (90°), a characteristic feature of a torsional plastic hinge.

#### 2.2.2. Hysteresis Curves

**Figure 9:**
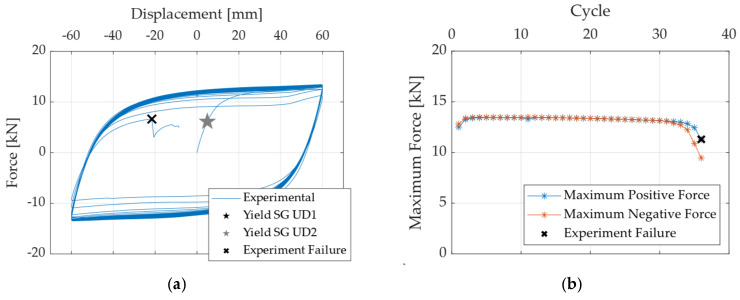
(**a**) Hysteresis curves obtained for specimen A572-0G-Fatigue1, and (**b**) maximum force recorded in each loading cycle.

Figure 9 shows the hysteresis curves obtained for specimen A572-0G subjected to the Fatigue 1 loading protocol, highlighting that the maximum force reached in each cycle remains almost constant after several cycles. The strain gauges (SGs) accurately captured the initial yielding point of the specimen.

**Figure 10:**
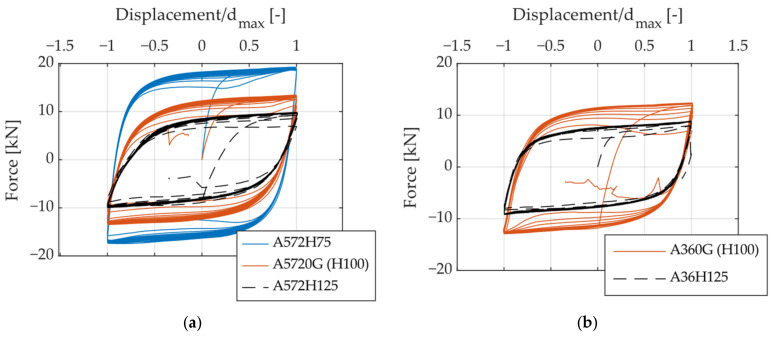
Hysteresis curves illustrating the effect of varying the height (H): (**a**) A572, (**b**) A36.

**Figure 11:**
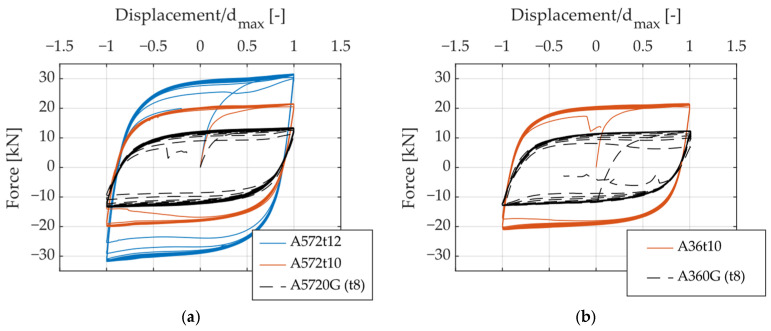
Hysteresis curves illustrating the effect of varying thickness (t): (**a**) A572, (**b**) A36.

**Figure 12:**
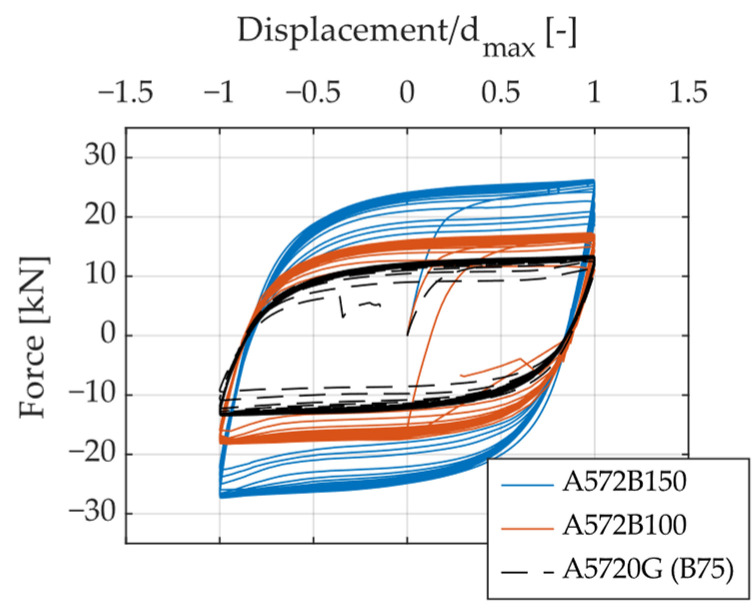
Hysteresis curves illustrating the effect of varying the width (B) of UFPs.

Figure 10, Figure 11 and Figure 12 present hysteresis curves illustrating the influence of specific geometric parameters on the cyclic behavior of UFPs. Displacement values in these curves were normalized by the maximum displacement reached in each specimen, facilitating direct and consistent comparisons among specimens. By varying the geometric parameters such as height (H), thickness (t), and width (B) of the UFPs, noticeable differences in cyclic response, specifically in stiffness and strength, were observed. A reduction in height resulted in increased stiffness and load-carrying capacity. Similarly, increases in thickness and width led to enhanced stiffness and strength. These observations underline the significant sensitivity of cyclic behavior to geometric variations, which can be effectively explained by Equation (1).

**Figure 13:**
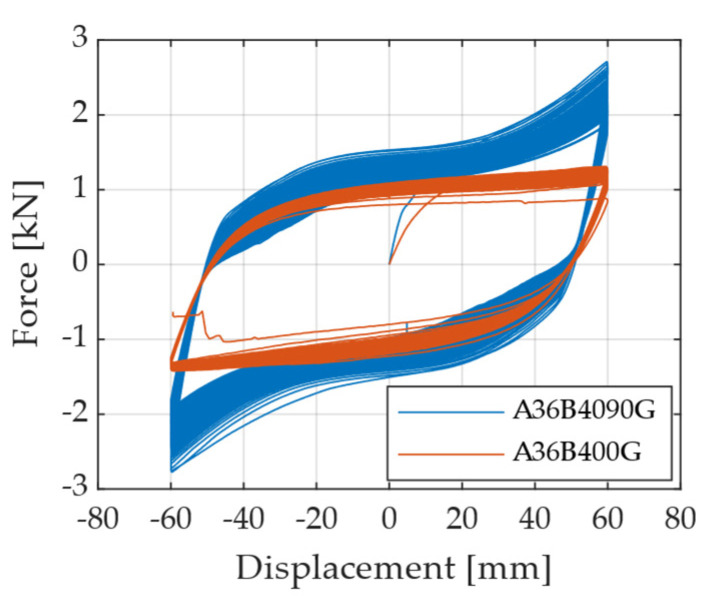
Hysteresis curves illustrating the effect of changing the direction of the applied displacement.

Figure 13 compares the force–displacement curves for specimen A572-B40 when subjected to loading directions of 0° and 90°. A significant change in the shape of the hysteresis loops is observed when the loading orientation is switched to 90°. Although both directions exhibit a clear linear-elastic region followed by yielding, the 90° loading condition demonstrates an increase in post-yield stiffness within the plastic region. This increased stiffness arises because, after the formation of torsional plastic hinges, the central portion of the curved segment elongates, causing the circular region to flatten. Consequently, the specimen experiences combined bending and axial stresses, resulting in increased resistance. These results highlight how loading direction influences the cyclic response and deformation mechanisms of the UFP elements.

#### 2.2.3. Yield and Ultimate Strength of UFPs

**Figure 14:**
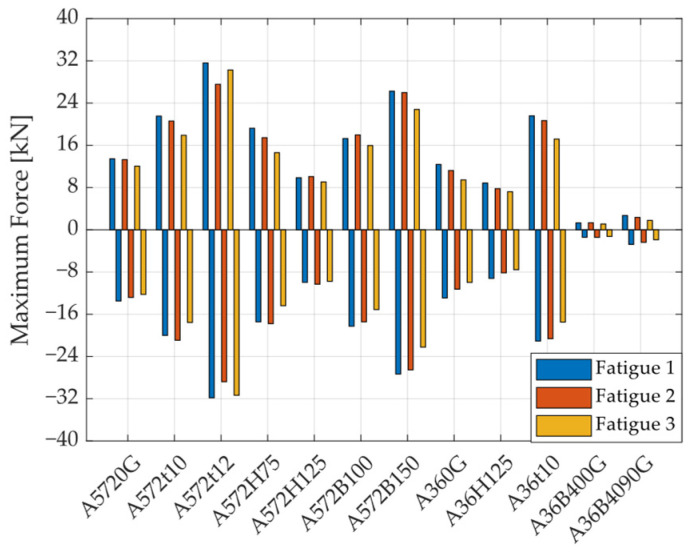
Maximum forces per specimen and loading protocol.

Figure 14 presents the maximum and minimum forces recorded for each specimen under the Fatigue 1, Fatigue 2, and Fatigue 3 loading protocols. The symmetry in the hysteresis curves for positive and negative load directions is clear from this graph. Additionally, the figure shows that forces do not significantly decrease between the Fatigue 1 and Fatigue 3 protocols, with differences lower than 20%. This indicates that, once the UFP elements reach displacement demands of around five times their yield displacement, they reach forces approaching their maximum capacity. Moreover, regarding the influence of steel type (A572 vs. A36), the results indicate that, in general, specimens fabricated from A36 steel exhibit slightly lower maximum forces across all fatigue protocols. This difference can be attributed to the inherent variation in tensile strength between the two materials: A36 steel typically ranges from 400 to 550 MPa, while A572 Grade 50 offers a higher range of approximately 450 to 620 MPa. As a result, A572 specimens demonstrate a moderately enhanced strength.

**Figure 15:**
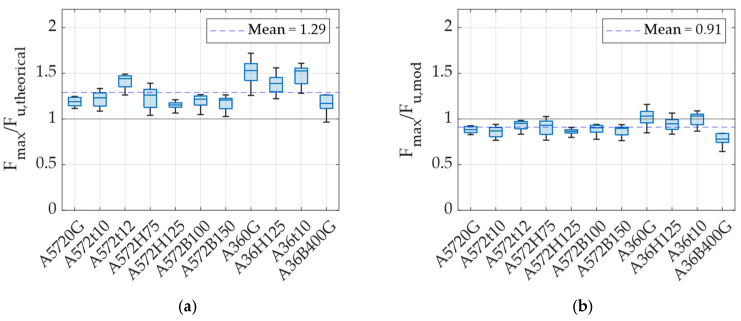
Ratio between the maximum force and the calculated force using (**a**) Equation (2) and (**b**) Equation (3).

Additionally, the experimentally obtained maximum force was compared with theoretical equations proposed by Deng et al. [24], developed to estimate the maximum force of UFP elements. Equation (2) corresponds to the maximum force generated by the UFP element when loaded at 0°, without considering isotropic strain-hardening effects, whereas Equation (3) accounts for this hardening. In Equation (3), the parameter $\sigma_{h}$ represents the difference between the ultimate and yield stresses, ν is the strain-hardening rate, and λ is a coefficient to be determined experimentally. In this study, λ takes the value of 5.025, following the work by Deng et al. [24]. Figure 15a presents the ratio of the experimentally measured maximum force for each specimen to the theoretical maximum force predicted by Equation (2). It is observed that, on average, experimental values are approximately 29% higher than theoretical predictions. This discrepancy occurs because Equation (2) does not include isotropic strain-hardening effects, thereby limiting the calculations to the yield stress ($f_{y}$). Nonetheless, this equation serves effectively as a conservative lower-bound estimate for the maximum force achievable by a UFP element. Notably, A36 specimens show greater dispersion than A572, likely due to the wider allowable variation in their tensile and yield strength.

(2) $$F_{u,\;theorical}=f_{y}\frac{Bt^{2}}{2\left(H-t\right)}$$

(3) $$F_{u,mod}=\left(f_{y}+\sigma_{h}\left(1-e^{-\nu \lambda t/H}\right)\right)\frac{{Bt}^{2}}{2\left(H-t\right)}$$

Figure 15b presents the ratio between the experimentally measured maximum force and the theoretical prediction obtained using Equation (3), which accounts for strain hardening. From this figure, it can be observed that experimental values are, on average, approximately 9% lower than theoretical predictions, representing a significant improvement compared to Equation (2). A possible explanation for this discrepancy is related to the specific values of parameters employed, which were directly adopted from the work of Deng et al. [24]. Ideally, these parameters should have been determined through an error minimization procedure tailored to the present experimental results. Nonetheless, Equation (3) with these parameter values effectively provides a reasonable upper-bound estimate of the maximum force capacity of UFP elements.

The yield point defines the transition between elastic and plastic behavior. In this study, the yield point was determined through a linear fitting approach based on the equation proposed by Ramberg and Osgood [34]. This method involves performing a fit using Equation (4), where d represents displacement, F is the force corresponding to displacement d, E is the elastic stiffness, and K and n are parameters obtained from the linear regression. Equation (4) can then be rearranged into Equation (5), where the term $EK^{-1/\left(n-1\right)}$ corresponds directly to the yield force. Subsequently, the yield displacement is determined by finding the displacement value on the elastic portion of the curve that corresponds to this yield force. The resulting yield point is thus considered an effective yield point since it does not necessarily lie on the original force–displacement curve obtained experimentally.

(4) $$d=\frac{F}{E}+K{\left(\frac{F}{E}\right)}^{n}$$

(5) $$d=\frac{F}{E}+\left(1+{\left(\frac{F}{EK^{\frac{-1}{n-1}}}\right)}^{n-1}\right)$$

**Figure 16:**
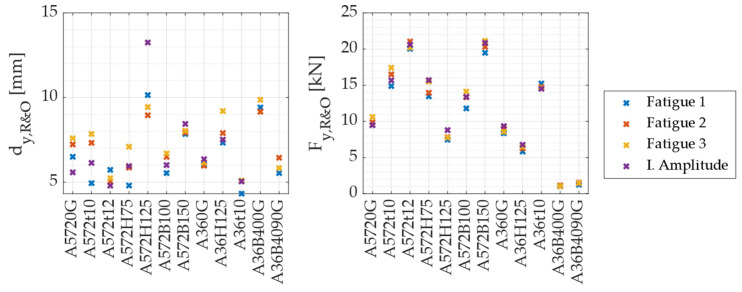
Yield displacement and yield force.

Figure 16 shows the values obtained for yield displacement and yield force for each specimen under the different loading protocols. These values clearly illustrate the influence of geometric parameters on yielding behavior. Specifically, the results indicate that an increase in plate thickness (t) leads, on average, to a decrease in yield displacement and a corresponding increase in yield force. Conversely, increasing the height (H) of the specimen results in greater yield displacement but lower yield force. Finally, regarding the width (B), a clear relationship with yield displacement could not be conclusively established: as the width increases from 75 to 100 mm, yield displacement decreases; however, further increasing B to 150 mm leads to an increase in yield displacement. Nevertheless, an increase in width (B) consistently results in an increase in the yield force.

The yield values can be compared with theoretical estimates proposed by Deng et al. [24] and Baird et al. [16] using Equations (6) and (7).

**Figure 17:**
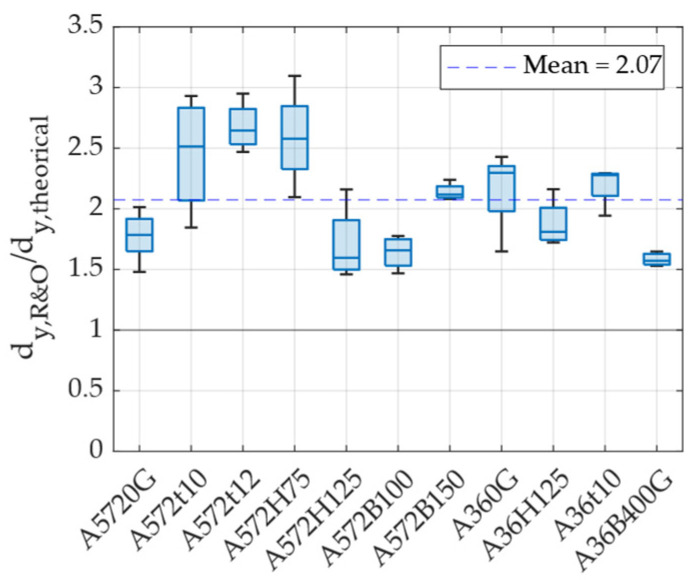
Ratio between the yield displacement obtained using the R&O method and the theoretical yield displacement calculated with Equation (1).

Figure 17 presents the ratio between the experimentally obtained yield displacement and the theoretical prediction for each specimen. The results indicate a significant discrepancy between experimental and theoretical values, with an average ratio of 2.07. One possible explanation for this difference is that the displacement calculated using Equation (1) corresponds to the first yield point, based on the yield force ($F_{y}$) value used in Equation (6). In contrast, the experimentally determined yield displacement corresponds to an effective yield point, which is located further from the origin compared to the initial yield point. Based on these findings, it can be concluded that, for UFP elements, the effective yield displacement (as determined using the Ramberg and Osgood approach) can be estimated by multiplying the theoretical yield displacement by a factor of 2, as expressed in Equation (7).

(6) $$F_{y}=f_{y}\frac{Bt^{2}}{3D}$$

(7) $$d_{y,R\&O}=\frac{27\pi F_{y}D^{3}}{8EBt^{3}}$$

**Figure 18:**
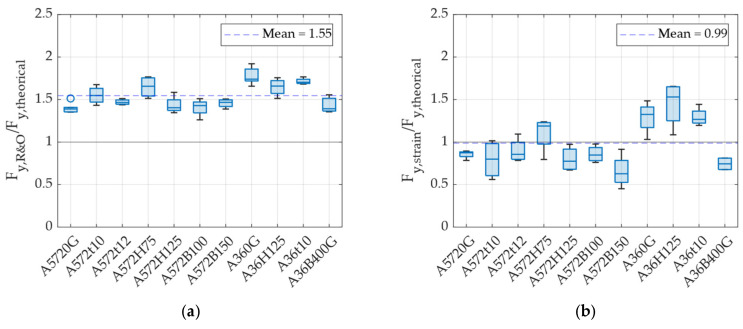
(**a**) Ratio between the experimental and theoretical yield forces, and (**b**) ratio between the yield force measured by strain gauges and the theoretical prediction.

Figure 18a shows the relationship between the experimental and theoretical yield forces. Similar to the previous figure, a discrepancy is observed between the compared values. However, in the case of yield forces, the experimental values are, on average, 1.55 times higher than the theoretical predictions. This difference arises from the same reason as before: the theoretical value corresponds to the first yield point, whereas the experimental value represents an effective yield point. To verify whether the theoretical yield force more accurately corresponds to the force at first yield, Figure 18b presents the ratio between the experimental yield force obtained from strain gauge measurements and the theoretical force calculated using Equation (6). The results indicate that, on average, the theoretical first yield force closely matches the experimentally measured first yield force. Based on these findings, Equation (8) is proposed to estimate the yield force using the Ramberg and Osgood approach, considering the geometric characteristics of a UFP element.

(8) $$F_{y,R\&O}=1.55\cdot f_{y}\frac{Bt^{2}}{3D}$$

#### 2.2.4. Displacement Ductility

**Figure 19:**
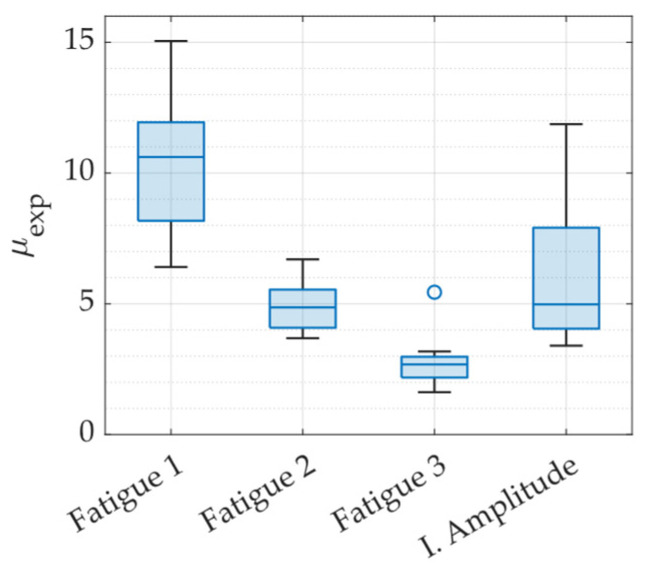
Displacement ductility achieved under each loading protocol.

Displacement ductility is a parameter commonly used in the design of energy dissipators for seismic-resistant structures. In this study, the experimental displacement ductility was calculated using the expression $\mu_{exp}=d_{máx}/d_{y,\;RO}$, where $d_{y,\;RO}$ represents the experimental yield displacement obtained using the Ramberg and Osgood (R&O) method, and $d_{máx}$ corresponds to the maximum displacement applied during testing. Figure 19 illustrates the distribution of ductility values achieved under each loading protocol. The results indicate that ductility varies across specimens depending on the applied protocol, which was expected, as the loading protocols were specifically designed to induce different ductility demands. It is worth noting that the target ductility for Fatigue 2 was set at 10, while for Fatigue 3, it was 5. The discrepancy between the expected and experimentally obtained ductility values arises from the method used to define the displacement amplitudes in each protocol. These amplitudes were based on the theoretical yield displacement calculated using Equation (1). As previously discussed, this theoretical value differs from the experimentally determined yield displacement obtained through the Ramberg and Osgood fitting approach, leading to variations in the final ductility measurements.

Furthermore, it is important to note that the ultimate displacement of the UFP elements could not be determined in any of the tests due to the maximum displacement limitations of the testing equipment. As a result, it was not possible to estimate the maximum ductility capacity of these energy dissipators, which may be limited by the length of the straight part of the UFPs. Despite this limitation, some specimens were subjected to displacement ductility demands exceeding 10, yet they continued to exhibit stable behavior throughout the tests. This result demonstrates that UFPs are an excellent choice for seismic-resistant applications.

#### 2.2.5. Low-Cycle Fatigue

**Figure 20:**
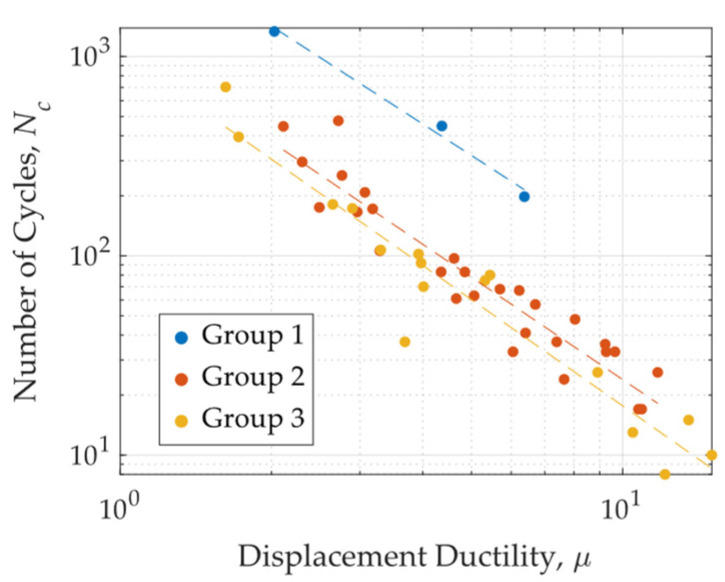
Number of cycles to failure versus displacement ductility for three H/t ratio groups.

Figure 20 presents the number of cycles to failure versus the displacement ductility achieved in each test, while also indicating the H/t ratio for each specimen. The specimens were categorized into three H/t ratio groups, as detailed in Table 2. The results reveal a clear relationship between displacement ductility demand and fatigue life. Specifically, as ductility demand increases, the number of cycles the specimen can withstand decreases exponentially. Additionally, a higher H/t ratio is associated with an increase in the number of cycles to failure. This trend aligns with findings from Deng et al. [24], who proposed that the fatigue life of a UFP element is strongly influenced by its H/t ratio. The connection between fatigue and displacement ductility arises from the deformation mechanisms involved. Higher ductility implies larger displacements, which in turn lead to a greater portion of the specimen undergoing plastic deformation, increasing accumulated damage. Conversely, as the H/t ratio increases, the deformation experienced by the most stressed fiber decreases, which helps extend the fatigue life of the specimen.

Based on the obtained results, exponential fitting curves were derived for each H/t ratio group, as presented in Equations (9)–(11), for groups 1, 2, and 3, respectively. In these equations, *N_c_* represents the number of cycles to failure, while *μ* denotes the achieved ductility. These equations are expressed in terms of displacement ductility, as this parameter is commonly used in the design of energy dissipators for seismic-resistant structures.

(9) $$N_{c}=4399\mu^{-1.63}$$

(10) $$N_{c}=1217\mu^{-1.71}$$

(11) $$N_{c}=1041\mu^{-1.77}$$

#### 2.2.6. Stiffness and Hysteretic Damping

**Figure 21:**
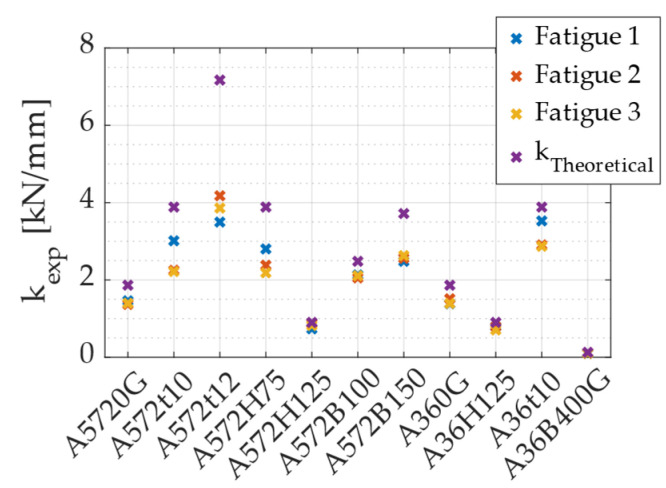
Stiffness per specimen.

The initial stiffness was determined as the slope of the initial linear portion of the force–displacement curve. To obtain this value, a linear regression was applied to selected data points within this region, ensuring that the displacement remained below 90% of the theoretical yield displacement. Figure 21 presents the stiffness values obtained for each specimen in each test, alongside the theoretical stiffness predicted by the equation proposed by Baird et al. [16], expressed in Equation (12). The results indicate that stiffness increases with greater width (B) and thickness (t) of the specimen, while increasing height (H) leads to a decrease in stiffness. For tests conducted at 90°, an increase in stiffness was observed compared to tests at 0°. However, since only one geometric configuration was tested in the 90° loading direction, it is not appropriate to establish a general trend based on this limited dataset.

(12) $$k_{Theoretical}=\frac{16EB}{27\pi }{\left(\frac{t}{D}\right)}^{3}$$

**Figure 22:**
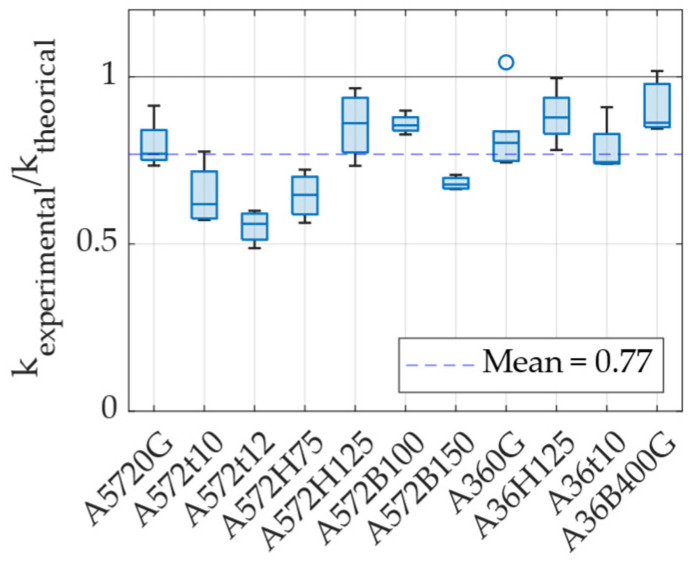
Ratio between experimental and theoretical stiffness.

Figure 22 shows the ratio between experimental and theoretical stiffness. On average, experimental stiffness was found to be 23% lower than the theoretical prediction given by Equation (12), with the A36 specimens showing results that more closely aligned with theoretical values. One possible explanation for this discrepancy lies in the manufacturing process of the UFP elements. These devices were cold formed using rollers to shape the curved section, a process that may have introduced residual stress and permanent deformations, potentially affecting the overall stiffness. Despite this difference, the average experimental-to-theoretical stiffness ratio can be used as a correction factor for estimating the actual stiffness of these specific UFP elements. Using this approach, Equation (13) allows for the estimation of the experimental stiffness of a UFP element loaded at 0° based on its theoretical stiffness.

(13) $$k_{exp}=0.77k_{theoretical}$$

**Figure 23:**
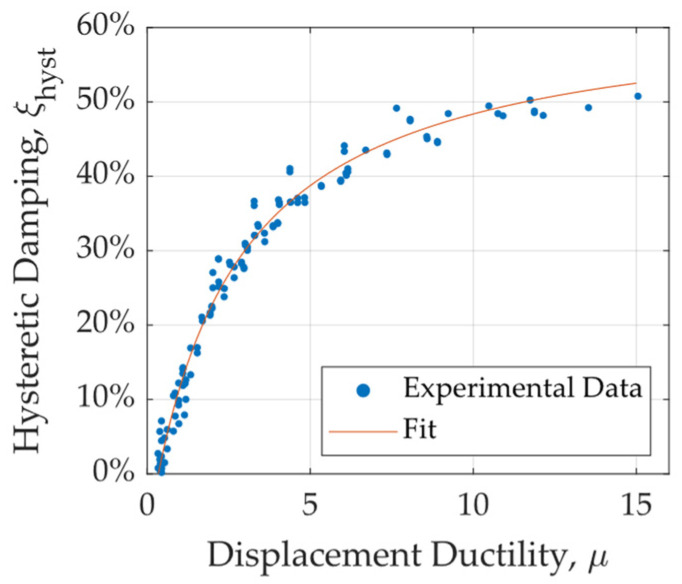
Hysteretic damping versus displacement ductility.

Hysteretic damping is a parameter used to quantify the energy dissipation capacity of a structure or system due to nonlinear behavior. It is computed using Equation (14), where $A_{d}$ represents the energy dissipated per cycle, while $F_{max,\;cycle}$ and $\delta_{cycle}$ correspond to the maximum force and displacement attained during a given cycle [35]. Figure 23 presents the hysteretic damping values obtained for different specimens under various ductility demands. The results indicate that UFP elements achieve damping values exceeding 45% as ductility increases. Additionally, for ductility levels greater than 5, damping consistently exceeds 40%, which is considered an appropriate level for the expected deformation demands in energy dissipation applications. To facilitate damping estimation, a rational curve fit was applied to the data, allowing for the prediction of hysteretic damping as a function of displacement ductility demand. The importance of this curve lies in its practical application for estimating the damping contribution of UFP dissipators at various ductility levels. The corresponding equation is presented as Equation (15), which can be utilized to estimate the added hysteretic damping provided by these devices during a displacement-based or performance-based seismic design of structures.

(14) $$\xi_{hyst}=\frac{1}{2\pi }\frac{A_{d}}{F_{max,cycle}\cdot \delta_{cycle}}$$

(15) $$\xi_{hyst}=\frac{\left(62.94\mu -21.42\right)}{\mu +2.57}$$

## 3. Numerical Characterization of UFPs

This section presents the development of finite element models (FEMs) using ANSYS [36], aiming to validate them against experimental results and to analyze the behavior of UFP elements subjected to different loading protocols.

### 3.1. Model Description and Calibration

**Figure 24:**
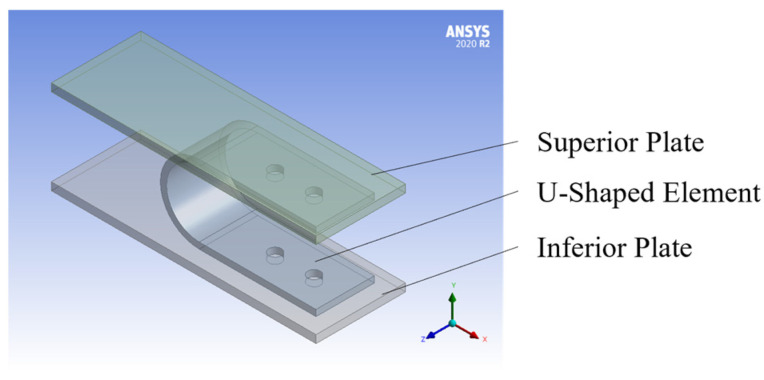
Finite element model geometry.

The finite element model was developed in ANSYS, consisting of three distinct volumes: one representing the UFP element and two additional volumes corresponding to constraining plates that restrict the displacement of the device, as illustrated in Figure 24. To accurately replicate the behavior of A36 and A572 steel, isotropic elasticity was assigned. Additionally, the model incorporated kinematic hardening using the Chaboche law (Equation (16)) [37] and isotropic hardening based on the Voce law (Equation (17)) [38]. In those equations, *α* corresponds to the shift of the material’s yield surface, *n* corresponds to the number of curves used to characterize the material, $C_{k}$ and $\gamma_{k}$ are model parameters, ${\bar{\varepsilon }}^{pl}$ is the equivalent plastic strain, $\sigma^{0}$ represents the updated yield stress due to hardening, ${\left.\sigma \right|}_{0}$ denotes the initial yield stress, $Q_{l}$ and $Q_{\infty }$ are the linear and exponential hardening coefficients, respectively, and ν is a saturation parameter. In this model, it was assumed that stress saturates at a constant value, and therefore, the value of $Q_{l}$ was taken as zero. The parameter $Q_{\infty }$ was defined as the difference between the ultimate strength ($f_{u}$) and the yield stress ($f_{y}$) while the parameter ν was varied during the calibration process.

Regarding boundary conditions, contact surfaces were defined between the outer faces of the UFP element and the inner surfaces of the restraining plates. Furthermore, the surfaces of the bolt holes were constrained to follow the displacement of the support plates. For meshing, second-order tetrahedral elements (Tet10) were used, ensuring a balance between computational efficiency and accuracy in the obtained results. The mesh size was optimized to minimize computational time while maintaining precision.

(16) $$\alpha =\sum_{k=1}^{n}\frac{C_{k}}{\gamma_{k}}\left(1-e^{-\gamma_{k}{\bar{\varepsilon }}^{pl}}\right)$$

(17) $$\sigma^{0}={\left.\sigma \right|}_{0}+Q_{l}{\bar{\varepsilon }}^{pl}+Q_{\infty }\left(1-e^{-\nu {\bar{\varepsilon }}^{pl}}\right)$$

**Figure 25:**
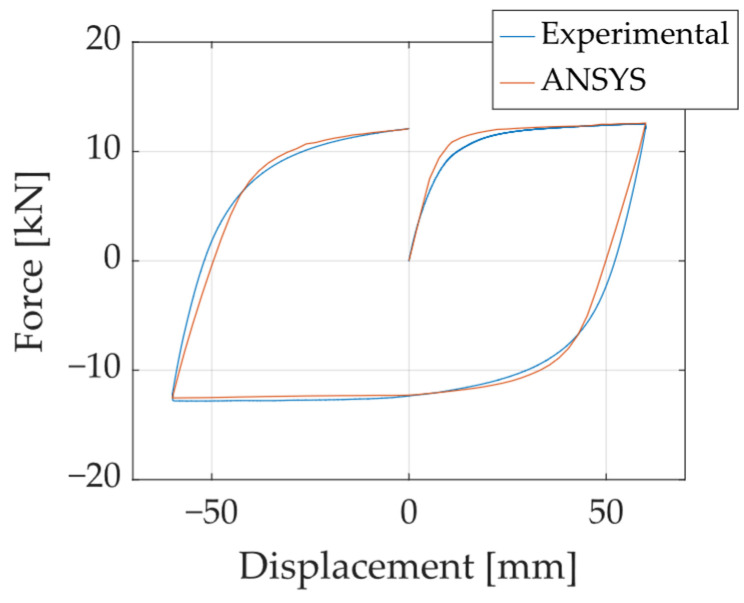
Comparison for the first cycle of the A572-0G-Fatigue1 test.

The parameters selected for calibration included specific coefficients from the Chaboche and Voce constitutive laws, as well as the elastic modulus of steel (E). For the calibration of A572 steel, the A572-0G specimen tested under the Fatigue 1 protocol (Figure 25) was used, whereas for A36 steel, the A36-B40-0G specimen under the Fatigue 1 protocol was utilized. The calibration metrics employed are defined in Equation (18), where $k_{sim}$ and $k_{exp}$ correspond to the simulated and experimental stiffness values, and $F_{sim}$ and $F_{exp}$ are the simulated and experimental force responses. This analysis yielded difference (DIF) values of 0.73% for A572 steel and 0.74% for A36 steel, along with root mean square error (RMSE) values of 7.6% and 13.8%, respectively. The final set of calibrated parameters is summarized in Table 3.

(18) $$DIF=\frac{\left|k_{sim}-k_{exp}\right|}{k_{sim}}\;\mathrm{and}\;RMSE=\sqrt{\frac{\sum {\left(F_{sim}-F_{exp}\right)}^{2}}{n}}/\sqrt{\frac{\sum F_{sim}^{2}}{n}}$$

**Figure 26:**
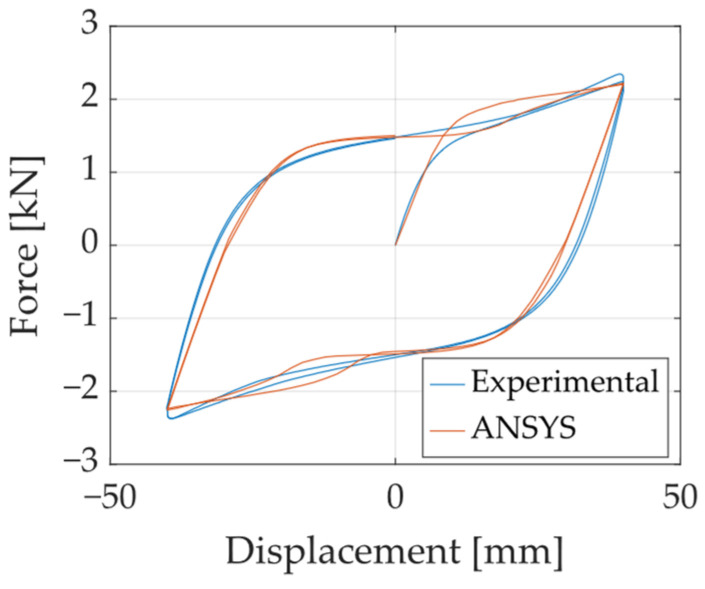
Experimental vs. numerical response for the A36-B40-90G-Fatigue2 test.

**Figure 27:**
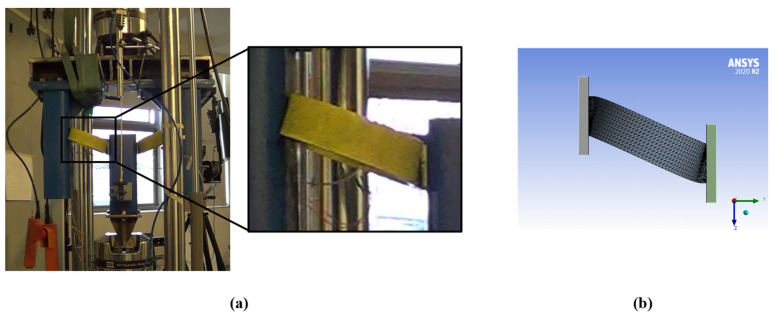
Comparison of deformed shapes in the 90° test: (**a**) Experimental, (**b**) Numerical.

Using the calibrated parameters, a numerical simulation was performed for a specimen loaded at 90° to evaluate the accuracy of the calibration. Specifically, the A36-B40-90G specimen subjected to the Fatigue 2 loading protocol was analyzed. The comparison of hysteresis curves is presented in Figure 26, while a comparison of deformed shapes is shown in Figure 27. These results confirm that the FEM effectively captures the cyclic behavior of UFP elements across different loading conditions, demonstrating its reliability for further numerical studies.

### 3.2. Influence of Loading Direction and Geometric Ratios on Stiffness and Yield Strength

This section proposes an equation to estimate the stiffness (*K*) and yield force (*F_y_*) of a UFP when subjected to 90° displacement loading. While Deng et al. [24] proposed a formula for estimating the ultimate strength under this loading condition, no further information is available regarding stiffness and yield force behavior. To address this gap, this study introduces a set of correction factors, as defined in Equation (19). These factors serve as multipliers to estimate the 90° stiffness and yield force from their corresponding values at 0°.

(19) $$K_{90{}^{\circ}}=C_{K}K_{0{}^{\circ}}\;,\;F_{y,\;90{}^{\circ}}=C_{F_{y}}F_{y,0{}^{\circ}}$$

The aim of this section is to determine the appropriate coefficients in terms of geometric parameters of UFP elements. To estimate these correction factors, independent variables were selected based on their influence on UFP behavior and deformation patterns. The chosen parameters include height-to-thickness ratio (H/t), width-to-thickness ratio (B/t), and effective length-to-width ratio (L’/B), where L’ is measured from the start of the curved section to the beginning of the bolted connection (see Figure 1). To ensure practical applicability, the working ranges for these parameters were established based on previous studies [9,10,17]: H/t between 8 and 16, L′/B between 1 and 3, and B/t between 6 and 12.5.

**Figure 28:**
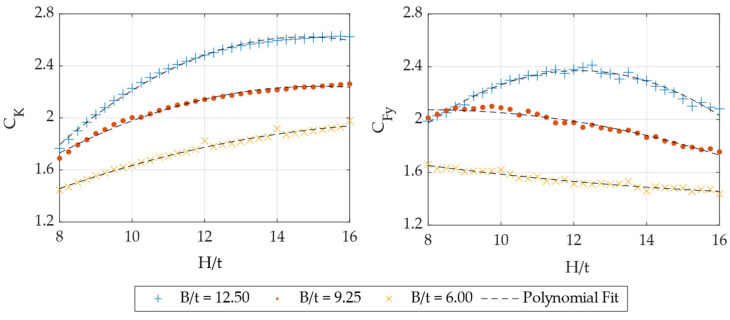
C_K_ and C_Fy_ factors for L’/B = 1.

To develop numerical models for determining $C_{K}$ and $C_{F_{y}}$, a set of FEMs was generated with varying geometric ratios. Since the objective is to construct lookup tables containing equations for estimating the correction factors, the selected variables were discretized as follows: L’/B at values {1; 2; 3}, B/t at values {6; 9.25; 12.5}, and H/t (being the most widely studied parameter) was discretized into 33 equally spaced values within the specified range. A total of 297 FEMs were generated, combining all possible values from the three parameter sets. The models assumed A572 steel as the material and were subjected to monotonic displacement loading at both 0° and 90°. The force–displacement curves obtained from each simulation were used to extract stiffness and yield force values, allowing the calculation of $C_{K}$ and $C_{F_{y}}$ for each set of geometric conditions. Figure 28 presents an example of the computed coefficients for L’/B = 1. The data revealed that these correction factors could be well-approximated using quadratic functions, enabling their estimation based on UFP geometric ratios. The general form of these equations is expressed in Equation (20), with the corresponding coefficients for each geometric condition provided in Table 4. It is important to note that these equations should only be applied within the specified validity ranges. If a required correction factor corresponds to an intermediate geometric ratio (L’/B or B/t) not listed in the table, a linear interpolation between the available values is recommended to obtain an initial estimate.

(20) $$C_{k}\;or\;C_{F_{y}}=a_{0}+a_{1}\frac{H}{t}+a_{2}{\left(\frac{H}{t}\right)}^{2}$$

### 3.3. Numerical Analysis of a Multidirectional System

**Figure 29:**
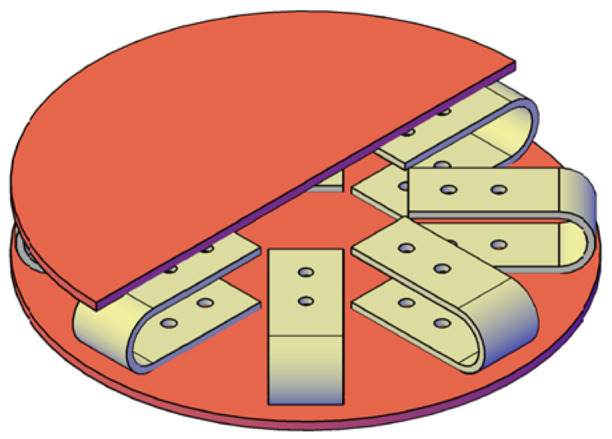
Isometric view of the multidirectional device based on UFP elements.

This section presents a multidirectional energy dissipation system based on UFPs, evaluating the influence of various parameters on the mechanical properties of the system. The system consists of two connection plates (upper and lower) and a defined number of UFP elements arranged in either a radial or axially symmetric configuration, as illustrated in Figure 29. This system was originally intended for bridge applications [6], specifically at the interface between the superstructure and substructure to limit excessive displacements. This system also demonstrates potential for broader seismic-resistant applications, including base-isolated buildings and other structures requiring enhanced energy dissipation.

**Figure 30:**
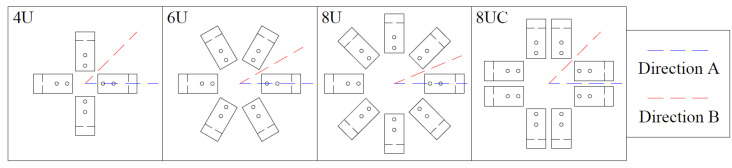
Proposed UFP element configurations for the multidirectional system.

To analyze the influence of these parameters, FEMs of the multidirectional device were developed. The modeling approach is identical to that of a single UFP element, with the primary difference being the expanded geometry, which includes multiple UFP elements and larger connection plates. The specimen used for these models corresponds to A572-0G. Figure 30 presents a top-view representation of the proposed configurations for the models.

The loading protocols consisted of two cycles of ±40 mm applied in a predetermined direction. Each model was subjected to this loading protocol in two directions, A and B, as indicated in Figure 30. Direction A is aligned with the axis of a UFP element, while Direction B is oriented between two adjacent UFP elements. These cases were analyzed to assess the multidirectional behavior of the system.

#### 3.3.1. Results and Discussion

**Figure 31:**
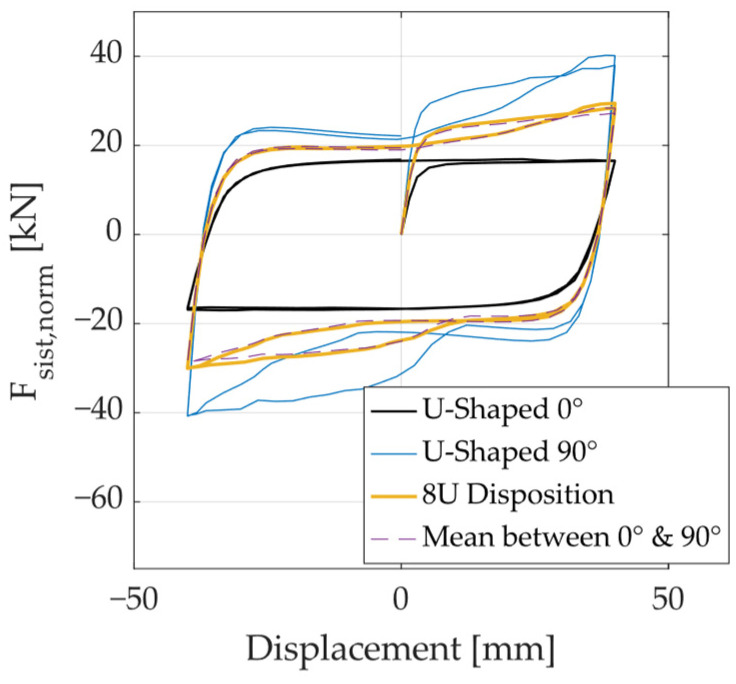
Force–displacement curve of a single element loaded at 0° and 90°, and the normalized response of the 8U configuration.

To facilitate comparison of the hysteresis curves, the system force was normalized by the number of UFP elements using the following expression: $F_{sist,norm}=F_{sist}/N_{u}$, where $F_{sist}$ is the total force resisted by the device and $N_{u}$ is the number of UFP elements. Figure 31 presents the hysteresis curve for the 8U configuration, along with the hysteresis of a single UFP element loaded at 0° and 90°, as well as their average response. The results show that the average response of a single UFP at 0° and 90° closely matches the response of the 8U configuration. This suggests that the overall behavior of a multidirectional energy dissipation system based on UFPs can be fully characterized by analyzing the response of a single UFP loaded at 0° and 90°.

**Figure 32:**
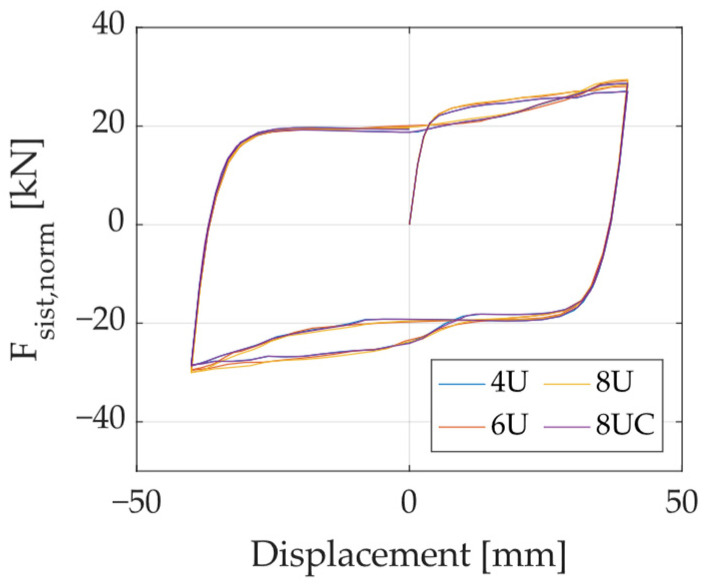
Normalized force–displacement curves for the different configurations loaded in Direction A.

Additionally, Figure 32 presents the hysteresis of all configurations under loading in Direction A. The results indicate that, regardless of the number or arrangement of UFP elements, the force per unit element remains the same when the loading direction aligns with a single UFP element.

Lastly, Table 5 summarizes the normalized maximum force attained by the different configurations under loading in Directions A and B, as well as the percentage difference between them. The findings suggest that, when the system consists of three or more UFP elements, its behavior remains practically identical in any loading direction.

#### 3.3.2. Proposed Procedure for Estimation of System Stiffness and Yield Force

**Figure 33:**
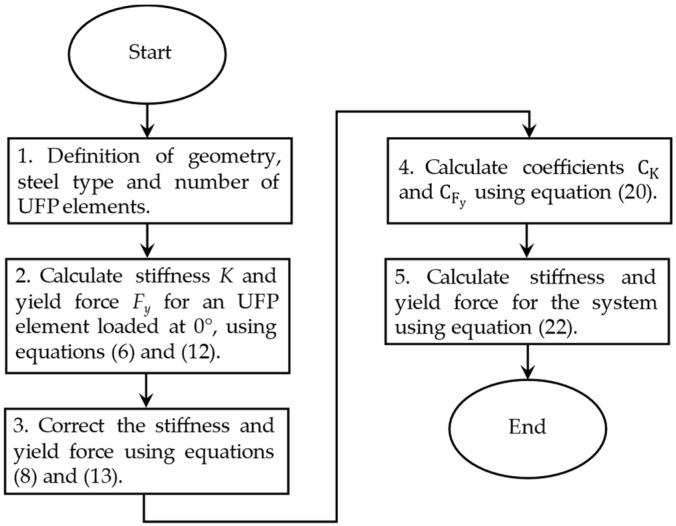
Flowchart for obtaining the stiffness and yield force of a multidirectional UFP system.

Based on the previous results and considering that the force–displacement response of the multidirectional device is solely dependent on the behavior of a single UFP loaded at 0° and 90°, a method for estimating system stiffness (*K_sist_*) and yield force (*F_y_*_,*sist*_) is proposed using Equation (21), where *N_u_* is the number of UFP elements in the system. As established in the previous section, both stiffness and yield force of a UFP loaded at 90° can be determined from its properties at 0°. Therefore, the mechanical properties of the entire system can be expressed exclusively in terms of the properties of a single UFP loaded at 0°, as presented in Equation (22). To facilitate the estimation of stiffness and yield force for the multidirectional dissipation system, a flowchart outlining the step-by-step procedure is provided in Figure 33.

(21) $$K_{sist}=\frac{\left(K_{0{}^{\circ}}+K_{90{}^{\circ}}\right)}{2}N_{u}\;;\;F_{y,\;sist}=\frac{\left({F_{y}}_{0{}^{\circ}}+{F_{y}}_{90{}^{\circ}}\right)}{2}N_{u}$$

(22) $$K_{sist}=K_{0{}^{\circ}}N_{u}\frac{1+C_{K}}{2}\;;\;F_{y,\;sist}={F_{y}}_{0{}^{\circ}}N_{u}\frac{1+C_{F_{y}}}{2}$$

## 4. Conclusions

This study investigated the cyclic behavior, fatigue life, numerical modeling, and system-level performance of UFPs for their implementation in multidirectional energy dissipation systems. A combination of experimental testing, numerical simulations, and parametric analyses was conducted to evaluate their mechanical properties and provide practical design recommendations. The findings led to the proposal of correction factors for effective yield estimation, validation of FEM techniques, and the development of a simplified formula to estimate the stiffness and yield force of UFP-based dissipation systems. These results contribute to improving the reliability and efficiency of seismic energy dissipation devices, facilitating their integration into engineering applications such as bridges and base-isolated structures. Based on the findings of this study, the following conclusions are drawn:

- The yield response of UFP elements was analyzed, revealing that existing formulas estimate the first yield point rather than an effective yield point that better represents their overall nonlinear response. To improve modeling accuracy, correction factors of 2 for displacement and 1.55 for force were introduced to adjust the theoretical yield values for bilinear or simplified models.
- The fatigue life of UFPs was found to be highly dependent on displacement ductility demand and the H/t ratio. Higher ductility demand led to a faster deterioration, while increasing H/t improved fatigue performance. These findings provide design guidance for optimizing UFP elements in applications requiring enhanced performance under cyclic loading.
- FEMs developed in ANSYS were validated against experimental results, confirming their accuracy in simulating UFP hysteresis behavior. When properly calibrated with kinematic and isotropic hardening models, the numerical approach successfully reproduced the stiffness, strength, and cyclic energy dissipation characteristics observed in the laboratory. This validation supports the use of FEMs for performance prediction and design optimization of UFP-based systems.
- The study also demonstrated that the number of UFP elements in a multidirectional dissipation system affects only the total force capacity, while the normalized force–displacement response remains unchanged. This observation enabled the development of a simplified estimation method, allowing designers to predict the stiffness and yield force of a multidirectional system using only the properties of a single UFP loaded at 0°.
- The proposed formula, combined with validated correction factors, provides a practical tool for engineers seeking to implement UFP-based dissipation devices in seismic-resistant structures. The findings contribute to advancing energy dissipation technology, promoting the widespread adoption of UFP elements in bridges, base-isolated buildings, and other structures requiring enhanced seismic resilience.

From a practical perspective, the results presented in this study provide insights into the seismic performance of UFPs, considering both material and geometric parameters. The experimental and numerical findings demonstrate the reliability and energy dissipation capacity of these devices under cyclic and fatigue loading, making them suitable for use in a variety of structural applications. Importantly, the equations proposed in this work are intended to support displacement-based or performance-based seismic design approaches, where controlling inelastic deformation and energy dissipation is critical. These formulations can be directly applied to the design of seismic protection systems in bridges, buildings, and other civil infrastructure or adapted to other structural materials (i.e., shape memory alloys) and configurations (i.e., multidirectional devices or structural braces) where ductility and low-cycle fatigue behavior are key design considerations.

While the proposed design methodology provides practical estimates for the strength, stiffness, and energy dissipation capacity of UFP dampers, some limitations should be acknowledged. The simplified equations are derived from specific experimental conditions and material parameters and may not fully capture the behavior of UFPs under other conditions. In particular, long-term durability concern, such as material aging, and potential corrosion were not explicitly addressed in this study. Additionally, the methodology assumes idealized boundary conditions and uniform material properties, which may differ from real-world applications where construction tolerances, thermal effects, and environmental exposure play a role. These limitations suggest that, while the proposed approach is useful for early-stage design and performance-based assessments, further refinement and calibration are needed for detailed design, especially in critical infrastructure.

It is worth noting that additional testing on full-scale devices should be conducted to validate the applicability of the proposed equations in real-world scenarios. Moreover, the use of other materials and the integration of these dampers into multi-degree-of-freedom systems, including bridge and building prototypes, should be explored through large-scale simulations and shake table testing. These efforts aim to further bridge the gap between experimental research and practical seismic design implementation.

## Figures and Tables

**Table 1:** Test matrix for the experimental program.

Specimen	Structural Steel	H [mm]	B [mm]	t [mm]	Repetitions
A572-0G	A572 Gr 50 $f_{y}=345\;MPa$ (minimum)	100	75	8	4
A572-t10		100	75	10	4
A572-t12		100	75	12	4
A572-H75		75	75	8	4
A572-H125		125	75	8	4
A572-B100		100	100	8	4
A572-B150		100	150	8	4
A36-0G	A36 $f_{y}=250\;MPa$ (minimum)	100	75	8	4
A36-H125		125	75	8	4
A36-t10		100	75	10	4
A36-B40-0G		120	40	5	3
A36-B40-90G		120	40	5	3
				TOTAL	46

**Table 2:** H/t ratio groups defined for low-cycle fatigue.

Group	H/t Range Values
1	[]20, +∞ [
2	]10, 20]
3	]0, 10]

**Table 3:** Parameters obtained from the calibration.

Parameter	A572	A36
$\gamma_{1}$ [-]	0.4	1
$C_{1}$ [Mpa]	60	160
ν	1	1
${\left.\sigma \right|}_{0}$ [MPa]	415	260
$Q_{l}$ [MPa]	0	0
$Q_{\infty }$ [MPa]	150	160

**Table 4:** Coefficient values for the equations describing C_K_ and C_Fy_**.**

Ratio		C_K_			C_Fy_		
L’/B	B/t	$a_{0}$	$a_{1}$	$a_{2}$	$a_{0}$	$a_{1}$	$a_{2}$
1	12.5	−1.31	0.53	−0.018	−1.01	0.56	−0.023
	9.25	−0.06	0.30	−0.010	1.74	0.08	−0.005
	6.00	0.36	0.17	−0.005	2.03	−0.06	0.001
2	12.5	−1.63	0.40	−0.012	−0.47	0.28	−0.008
	9.25	−0.95	0.31	−0.010	−1.03	0.43	−0.016
	6.00	−0.15	0.16	−0.005	0.40	0.17	−0.006
3	12.5	−1.10	0.24	−0.006	−0.24	0.17	−0.003
	9.25	−0.95	0.24	−0.007	−0.30	0.21	−0.005
	6.00	−0.40	0.15	−0.004	−0.32	0.23	−0.007

**Table 5:** Maximum force difference under loading in Directions A and B.

	Maximum Force [kN]		Difference [%]
Configuration	Direction A	Direction B	
4U	28.7	30.4	6.1
6U	29.2	30.0	2.7
8U	29.4	29.6	0.6
8UC	28.6	30.3	5.9

## Data Availability

The raw data supporting the conclusions of this article will be made available by the authors on request.
